# Bone Defects in Revision Total Knee Arthroplasty and Management

**DOI:** 10.1111/os.12425

**Published:** 2019-02-27

**Authors:** Peng‐fei Lei, Ru‐yin Hu, Yi‐he Hu

**Affiliations:** ^1^ Department of Orthopaedic Surgery Xiangya Hospital of Central South University Changsha China; ^2^ Department of Orthopaedic Surgery Guizhou Provincial People's Hospital Guiyang China

**Keywords:** Bone defects, Knee joint pain, Management, Revision of total knee arthroplasty

## Abstract

This article reviews the recent updates in revision of total knee arthroplasty (RTKA). We reviewed the recent articles on RTKA in databases including PubMed, Google Scholar, and SCOPUS. Total knee arthroplasty (TKA) involves the replacement of all three compartments of the knee in surgery of the knee joint to restore capacity and function. TKA is one of the most common and reliable surgical treatment options for the treatment of knee diseases. However, some patients require revision of TKA (RTKA) after primary TKA for various reasons, including mechanical wear, implant loosening or breakage, malalignment, infection, instability, periprosthetic fracture, and persistent stiffness. Unfortunately, the overall outcome of RTKA is not as satisfactory as for primary TKA due to the uncertainty regarding the actual success rate and the risk factors for failure. Cementation, modular metal augmentation, bone grafting, autologous bone grafting, allogenic bone grafting, impactation bone grafting, structural bone allografting, metaphyseal fixation, using porous titanium coated press fit metaphyseal sleeves and porous tantalum structural cones, and megaprostheses or customized prostheses are the currently available management options for RTKA. However, most of the management systems possess specific complications. Novel approaches should be developed to improve functional capacity, implant survival rates, and quality of life in a cost‐efficient manner.

## Introduction

Knee arthroplasty is a surgical procedure undertaken to replace the weight‐bearing surfaces of the knee joint to restore capacity and function^1^. It can be performed as a partial, also called unicompartmental arthroplasty (UKA, which replaces only the damaged surfaces retaining any undamaged parts) or a total knee arthroplasty (TKA)^2^, ^3^. TKA involves replacement of all three compartments of the knee, known as the medial compartment (inside aspect of the knee), the lateral compartment (outside of the knee), and the patellofemoral compartment (the joint between the patella and the femur)^4^. TKA is one of the most common and reliable surgical treatment options for the treatment of knee diseases such as rheumatoid arthritis, osteoarthritis, and osteonecrosis^5^. The goal of TKA is to reduce pain, enhance functional capacity, and improve health‐related quality of life and life expectancy^6^. TKA is a highly cost‐effective procedure and the incidence of TKA has increased dramatically in the aging population with knee arthritis over the past few decades.

Despite high success rates, many TKA patients remain dissatisfied with the clinical outcome as they develop chronic pain following TKA, which is a major health burden for them^7^. In addition, a significant number of patients require revision TKA (RTKA, a procedure that replaces the previously implanted artificial knee joint or prosthesis with a new prosthesis) after primary TKA for various reasons, including mechanical wear (such as polyethylene wear or metal wear), implant loosening or breakage, malalignment, infection, instability, periprosthetic fracture and persistent stiffness^6^, ^8^. However, the overall outcome of RTKA is not as satisfactory as primary TKA due to the uncertainty regarding the actual success rate and the risk factors for failure. It is reported to be an unenviable task that requires adequate exposure, implant extraction and restoration, and correction of bone loss, joint stability and soft tissue insufficiency to provide a stable and durable knee joint reconstruction^9^, ^10^.

Petersen *et al*. reported that patients with osteoarthritis (OA) undergoing RTKA experienced more chronic complications after surgery. In their study, 99 OA patients were investigated after RTKA surgery and found to have reduced function, poorer quality of life, and higher pain intensity compared to TKA patients^11^. Stambough *et al*. investigated a clinical study with 76 patients following RTKA and reported that as many as one‐third of patients had experienced complication or failure^12^. RTKA is reported to be associated with bone defects that can be caused by stress shielding, infection, osteolysis, mechanical bone loss generated from a loose implant or iatrogenic loss during implant removal^13^, ^14^. In this review, we discuss the bone defects following RTKA along with the potential appropriate management and treatment.

## Search Method

**Figure 1:**
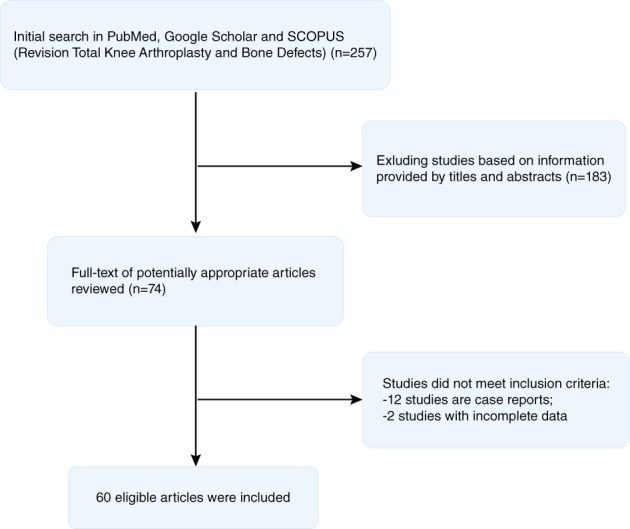
The selection flow for included studies in this review. The first screening step is based on information provided by titles and abstracts, and 183 articles were excluded. The second screening step is to exclude the case report studies (*n* = 12) and the studies with incomplete data (*n* = 2). Finally, 60 articles were included.

We have reviewed the recent articles on RTKA in databases including PubMed, Google Scholar, and SCOPUS. There were 257 articles and 183 were excluded based on titles and abstracts. Another 14 articles were excluded because they did not meet the inclusion criteria, with 12 studies that were case reports and 2 studies with incomplete data. Finally, 60 articles were included. The literature selection process is shown in Fig. 1.

## Classification of Bone Defects in Revision of Total Knee Arthroplasty

The main goal of RTKA is to relieve pain and improve function; however, this procedure is much more complex and difficult than primary TKA. RTKA has a greater risk of bone defects localized in areas corresponding to the tibia and femoral articular surfaces^8^, ^15^.

**Figure 2:**
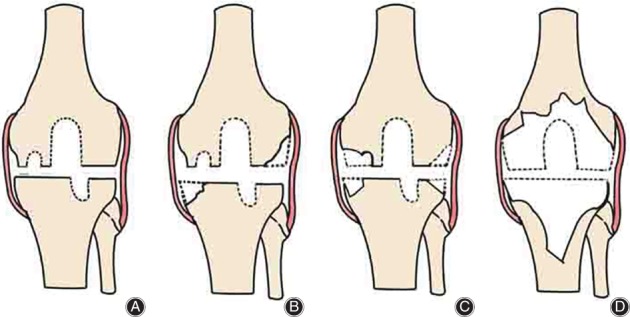
Anderson Orthopaedic Research Institute classification of bone defects: (A) type I (intact metaphyseal bone with minor defects not compromising the stability of a revision component), (B) type IIA (damaged metaphyseal bone with defects in one femoral condyle or tibial plateau), (C) type IIB (more than one damaged metaphyseal bone), and (D) type III (deficient metaphyseal bone with bone loss compromising a major portion of the condyle or plateau). The latter defects are occasionally associated with collateral or patellar ligament detachment and usually require bone grafting or custom implants^17^.

There are various classification systems of bone defects that are mainly based on size, severity, and location of the defects that enable accurate preoperative planning for management, predict outcomes, as well as provide guidelines on treatment and rehabilitation^15^, ^16^. Among these classifications, the Anderson Orthopedic Research Institute (AORI) classification is the most practical and frequently used system which predominantly depends on the size of the bone defect originated from the tibia and femur^17^. According to the AORI classification, bone defects are classified into three types for tibia (T1, T2, T3) and femur (F1, F2, F3) separately^18^. In type 1 (T1 and F1) defects, there is a minor bone defect without compromising the stability of a revision component. The development of posterior condyles remains normal. In type 2 (T2 and F2) defects, metaphyseal bone damage and cancellous bone loss occurred in one femoral condyle/tibial plateau (type 2A: T2A and F2A) or both femoral condyle/tibial plateau (type 2B: T2B and F2B). The development of the posterior condyles and/or tibial component is reduced. In type 3 (T3 and F3) defects, there is significant cancellous metaphyseal bone loss compromising the ligamentous instability of a majorportion of the condyle or plateau (Table 1) (Fig. 2)^15^, ^17^.

**Table 1:** The Anderson Orthopedic Research Institute classification of bone defects in revision of total knee arthroplasty^15^, ^16^, ^17^

Type	Severity of bone defects in tibia (T) and femur (F)
Type 1 (T1 and F1)	Minor bone defect without compromising the stability of a revision component, normal development of the posterior condyles
Type 2A (T2A and F2A)	Metaphyseal bone damage and cancellous bone loss in one femoral condyle/tibial plateau, reduced development of the posterior condyles, requiring reconstruction to maintain implant stability
Type 2B (T2B and F2B)	Metaphyseal bone damage and cancellous bone loss in one or both femoral condyle/tibial plateau, reduced development of the posterior condyles, requiring reconstruction to maintain implant stability
Type 3 (T3 and F3)	Significant cancellous metaphyseal bone loss compromising the ligamentous instability of a major portion of the tibial or femoral condyle, association with patellar tendon detachment

Revision of TKA often requires long diaphyseal supporting stems and the reconstruction of the tibial side is relatively more demanding than the femoral side. Mihalko *et al*. investigated a cohort of 120 RTKA patients with a diaphyseal slotted stem in which 20 (more than 16%) patients reported stem pain on the tibia^19^. Sierra *et al.* reported a study in which more than 80% of the 1814 index RTKA was localized on the tibial, femoral or both components, and 373 knees were at substantial risk of subsequent re‐revisions^20^.

## Preoperative Radiographic Diagnosis

Bone defects frequently occur in RTKA, which may be a consequence of the primary TKA. In primary TKA, the bone defects based on preoperative radiography are often underestimated relative to the actual bone defect found during RTKA^13^. Reish *et al*. report that plain radiography is inadequate for the detection of osteolytic lesions in TKA^21^. In their study, radiographic diagnosis was made for 31 patients with TKA. They detected only 8 osteolytic lesions by using plain radiographs while multi‐detector computed CT detected 48 osteolytic lesions in 31 knees, providing the more accuracy in diagnosis^21^. Thus, careful diagnostic and preoperative planning tools are essential during primary TKA.

## Management of Bone Defects in Revision of Total Knee Arthroplasty

Various techniques have been developed to manage the bone defects in RTKA, including cementation, modular metal augments, elimination of bone defects, bone grafts (autografts, allografts and structural massive bone allografts), metaphyseal fixation (porous titanium metaphyseal sleeves and porous tantalum metaphyseal cones), and megaprostheses (customized prostheses) (Table 2)^13^, ^15^.

**Table 2:** Management of bone defects in revision of total knee arthroplasty

Technique	Type of bone defect (according to Anderson Orthopedic Research Institute system)	Effects/advantages	Complications/disadvantages
Cementation^13^, ^15^	Type 1	Inexpensive, simple, and reproducible	Thermal necrosis, loosening, radiolucent lines
Modular metal augmentation^13^, ^15^, ^22^, ^23^	Types 2 and 3	Stable and durable, improves stability for components fixation	Fretting, radiolucent lines, corrosion, loosening
Autologous bone grafting^24^, ^25^	Type 1	Effective restoration of construct stability	Only used in small defect
Allogenic bone grafting^15^, ^26^	Type 1	Improves survival rate	Transmission of viral diseases, immunological reaction, and increased risk of infection
Impaction bone grafting^13^, ^27^, ^28^	Types 1 and 2A	Bone graft incorporation, improves construct stability	Resorption
Structural bone allograft^29^, ^30^, ^31^	Types 2 and 3	Improves mechanical stability for components fixation	Instability, fracture of the graft, transmission of bacterial and viral disease
Porous titanium metaphyseal sleeves^32^, ^33^, ^34^	Types 2 and 3	Improves construct stability and survival rate	Lack long‐term follow up
Porous tantalum metaphyseal cones^35^, ^36^, ^37^	Types 2 and 3	Provides structural and mechanical support, restoration of construct stability	Lack long‐term follow up, extraction difficulty, and fracture of the host bone
Megaprosthesis/customized prosthesis^15^, ^38^, ^39^	Types 2 and 3	Bioactivity	Expensive, poor versatile, delay to manufacture, short‐term mechanical complications, and infection

## Cementation

**Figure 3:**
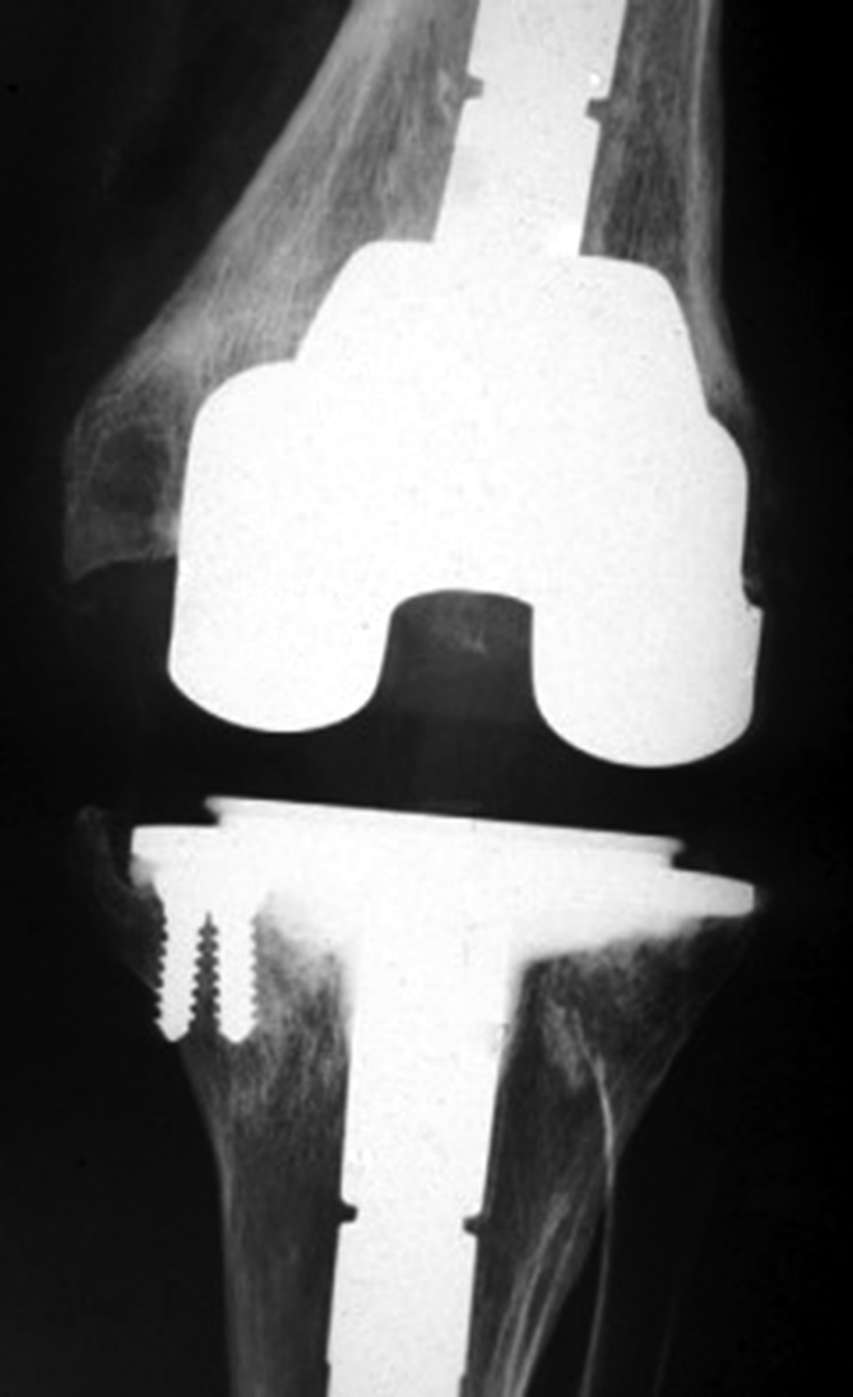
Postoperative X‐ray for revision total knee arthroplasty using screw‐reinforced cement technique at the tibial side; with lateral translation at the femoral side.

Cementation is an inexpensive, reliable, reproducible, and easily performed method. The use of cement, either alone or in combination with screws, is recommended for small bone defects (with defects less than 5 mm of depth) such as AORI type 1. This technique is not recommended for larger defects due to risk of thermal necrosis and loosening^13^. In cases of larger defects, a large volume of cement is used, leading to the release of a greater amount of heat, which may cause thermal necrosis as well as shrinkage of the volume of cement during polymerization and lamination. The results of cementation alone or with the support of screws are not satisfactory and this technique is used less due to the risk of loosening and frequent radiolucency in the bone–cement interface (Fig. 3)^15^. Lotke used cement alone in 59 knees with a defect of 10 to 20 mm (*n* = 33) or >20 mm (*n* = 23) in height and followed up for 7.1 years^22^. Forty‐three knees are non‐progressive in radiolucent lines, but only one failed and needed revision. Long‐term results of cement filling are good when the bone defects are <20 mm^23^. Dorr used cement alone in 54 patients with AORI type 1 defects. Patients were followed up for 7 years and only 1 had loosening and non‐progressive radiolucent lines. Ritter used cement with screws in 57 patients with tibial defects of 9 mm in mean height After a minimum of 3 years of follow‐up, 25% had non‐progressive radiolucency at the bone–cement interface, but none of the components failed. In ongoing follow up for 7 years, there was no progression of radiolucency lines in either the bone–cement or the cement–prosthesis interface^40^.

## Modular Metal Augments

**Figure 4:**
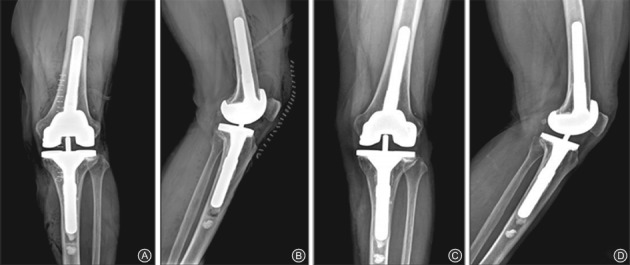
(A, B) Postoperative X‐ray for revision total knee arthroplasty using modular metal augments for bone defects; (C, D) 25 months after revision total knee arthroplasty using modular metal augments for bone defects, there is no radiolucent line.

Modular metal augmentation provides more stable and durable knee revisions with limited peripheral bone defects up to 20‐mm deep (AORI type 2 or 3 defect). Various types of augments (such as rectangular blocks and wedges) are available that allow selective augmentation for both femoral and tibial defects^13^, ^29^. The preference between wedges and blocks depends on the shape of bone defects. The augment that best fills the defect and, at the same time, removes little intact host bone should be used. Augments are screwed or cemented to fit the femoral and tibial components^15^. On the tibial side, modular metal wedges are usually used to augment the tibial tray for tibial bone stock deficiency. Femoral defects most often occur on the posterior surfaces and metal blocks can be used to increase femoral component rotation and to maintain the balance between flexion and extension gap. This technique should be reserved for elderly and less active patients^13^, ^15^. Metal augments confer the risk of some complications, including fretting, radiolucent lines and corrosion. It has also been reported that the metal augmentation technique may also cause the disassociation of modular components, leading to stress shielding and increased potential bone loss^41^. Werle *et al*. suggest that metal augmentation is an acceptable technique^24^. In their study, they used large (30 mm) metal distal femoral augments to compensate for type 3 bone defects and observed no radiographic evidence of loosening; no implants had been revised after a mean of 37 months. Patel *et al*. treated a total of 102 RTKA patients (type 2 defects) with metal augments and observed 92% survival at 11 years, with no significant complications, including fretting and loosening^41^. Lee *et al*. followed up 37 patients (39 knees) for more than 2 years after revision of infected TKA using modular metal augments for bone defects and concluded that increased modularity can result in radiological stability (Fig. 4)^25^.

## Bone Grafts

Bone grafting is used for the treatment of bone defects and enables restoration of the living bone stock. There are several types of bone grafting, such as impaction bone grafting, autografts, allografts, and structural massive bone allografts^26^, ^42^.

### *Autologous Bone Grafting*

Autologous bone grafting is a technique by which bone is harvested from non‐essential bones such as from the iliac crest and transplanted in the same individual to fill space and produce an osteogenic response in a very small bone defect^27^. Liu *et al*. use single or double distal femoral osteotomic bone to reconstruct the bone defects using hollow nail internal fixation. The Knee Society Score (KSS) was recorded, and patients were followed up for 6 to 9 years to investigate the efficacy of autologous bone grafting plus screw fixation for medial tibial defects. Effective restoration of knee mechanical axis and stability was observed, and the preoperative and postoperative KSS scores had significant differences in each group (*P* < 0.05)^28^. Batmaz *et al*. retrospectively reviewed 288 patients with primary bicompartmental knee arthroplasty who were operated on between April 2012 and June 2015^43^. Two groups were formed according to whether or not sealing of femoral tunnel with autologous bone graft was undertaken. An independent sample *t*‐test was used to compare the two groups. The results showed that the postoperative lowest hemoglobin levels were higher in the plugged group (*P* < 0.001), and drain outputs were much lower than in the unplugged group (*P* < 0.001). There is no statistically significant difference between the amount of given erythrocyte suspensions. The authors concluded that autologous bone grafting is a free to use, non‐time consuming, and effective method to reduce blood loss.

### *Allogenic Bone Grafting*

**Figure 5:**
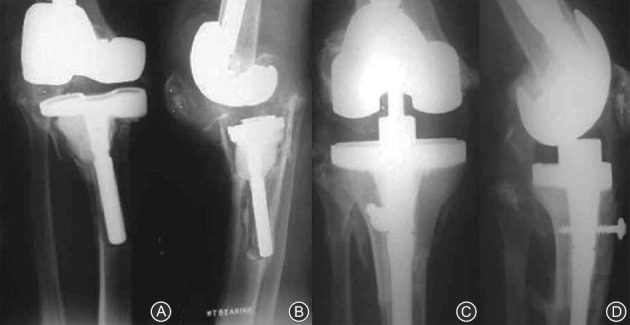
Preoperative anteroposterior (A) and lateral (B) radiographs of a right knee demonstrating an Anderson Orthopedic Research Institute type 3 tibial defect. Postoperative anteroposterior (C) and lateral (D) radiographs showing a revision with tibial allograft–implant composite. The tibial tubercle osteotomy is reattached to the allograft with a screw.

In this technique, the bone is harvested from an individual other than the one receiving the graft. Allograft can be taken from living and dead donors as fresh‐frozen or freeze‐dried bone allograft typically sourced from bone banks^44^. Bone allografts are reported to be associated with numerous risks, including transmission of viral diseases, immunological reaction, and increased risk of infection^15^. Franke *et al*. used allograft bone in 30 RTKA (27 patients) between 1994 and 2009. Patients were followed for a mean of 5 years, evaluated by KSS and radiological results in addition to review of complications. Predicted survivorship at 5 years as 93%, with further revision surgery as the end point. The average KSS was 76.4, with 19 (63%) knees scoring “excellent” results, 4 (14%) “good,” 1 (3%) “fair,” and 6 (20%) “poor” (Fig. 5)^45^. Their study demonstrated that the use of allograft bone in revision total knee replacement will result in significant  bone loss in the short to medium term.

### *Impaction Bone Grafting*

Impacted morselized bone grafting is an effective method that provides more durable and versatile restoration of living bone stock, especially in younger patients, for the management of contained (central form) and uncontained (peripheral) defects in RTKA^46^. In this technique, the morselized bone grafts are tightly packed around an implant by the addition of intramedullary stems and the implants are cemented with cooled antibiotic loaded bone cement. Metal wire mesh can also be used to form a more stable construct^47^. Impacted morselized bone grafts can be remodeled and incorporated with the host in reaction to surrounding loading pressures^30^. It is important for impacted bone grafting to maintain a balance between initial stability and long‐term incorporation. Lack of biomechanical support decreases initial stability, which may lead to RTKA failure before the eventual incorporation of the grafts^31^.

**Figure 6:**
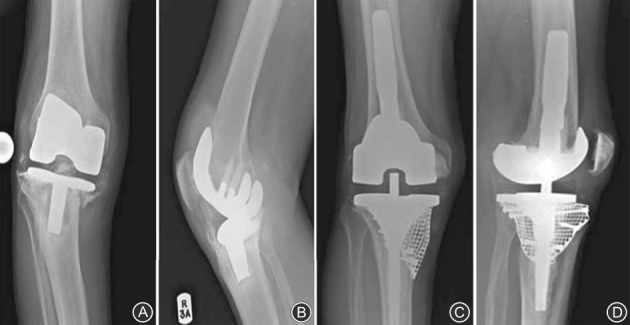
(A, B) Preoperative radiograph anteroposterior view and lateral view of a large tibial bone defects with instability. (C, D) Postoperative radiograph anteroposterior view and lateral view of short cemented stem with impacted bone graft for substantial tibial bone loss.

Numerous studies used impacted bone graft and suggested that additional supports are necessary with impacted bone graft in RTKA patients to achieve a stable implant^26^, ^48^. The use of intramedullary stems with impacted bone graft may provide an effective method to manage large bone defects in RTKA^46^. Naim *et al*. used a short cemented stem with impacted bone graft for substantial tibial bone loss (Fig. 6)^49^ **.** Minimum follow‐up was 2 years. The KSS improved from 27.4 to 89.2 on average, with Knee Society Function score and WOMAC increasing by 26.3 and 23.2 points, respectively. The observed stable construct provided excellent durability and versatility at 2 years. Lonner *et al*. review the results of 17 revision total knee arthroplasties in 14 patients in whom large uncontained defects were treated with impaction allografting and molded wire mesh for containment^50^. KKS increased from an average of 47 points to 95 points and function scores increased from 48 points to 73 points at the most recent follow up, which suggested that the use of impaction grafting with wire mesh is an effective method to treat massive uncontained bone loss in RTKA.

### *Structural Bone Allografts*

Structural bone allograft is relatively cost effective and provides stable and durable restoration of bone stock in large bone defects (AORI types 2 and 3)^42^. This technique offers potential for ligament and tendon reattachment and avoids unnecessary removal of host bone. It is important to properly select the type and size of allograft and the implant to fit the defect precisely^51^. Femoral heads, bulk distal femoral segments, and proximal tibial segments are most commonly used as structural allografts in RTKA to improve mechanical stability for component fixation. The use of intramedullary stems with sufficient length is recommended to provide some stress protection to the structural allograft^52^. However, the use of structural bone allograft is reported to be associated with numerous complications in RTKA, including instability, fracture of the graft, and the risk of bacterial and viral disease transmission^51^.

**Figure 7:**
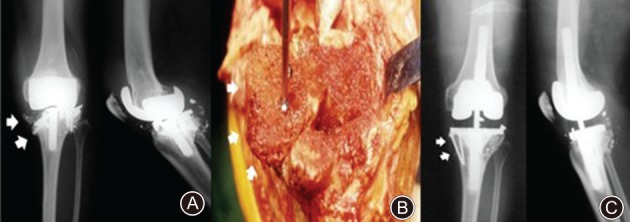
(A) Anteroposterior and lateral radiographs show a severe osteolysis in proximal tibia and distal femur with metal breakage (white arrow). (B) The femoral head allograft was stabilized with screws at the proximal tibia (white arrow). (C) The allograft remained intact with minimal resorption at 6 years after surgery (white arrow).

Several studies have demonstrated that the use of femoral head structural allograft might improve the clinical outcomes for patients with severe bone defects in RTKA^9^, ^32^. A prospective case series from the Royal London Hospital demonstrated that the use of femoral head structural allograft along with long stemmed components can achieve a successful result in the management of severe bone loss during RTKA^33^. Chun *et al*. assessed the mid‐term to long‐term clinical and radiographic results for severe bone defects of 27 patients undergoing RTKA using a fresh frozen femoral head allograft and a standard condylar implant with a diaphyseal‐engaging stem (Fig. 7)^53^. In their study, 26 out of 27 knees were observed to have no collapse, disease transmission or stress fractures, and the mean range of motion had increased from 71° to 113° and the mean Hospital for Special Surgery knee score had improved from 46 to 83 points, providing a reliable and durable result.

## Metaphyseal Fixation

Metaphyseal fixation improves the construct stability during the treatment of severe bone defects in RTKA. Metaphyseal fixation with porous‐coated sleeves and trabecular metal cones has documented good survival at short‐term follow up^34^.

### *Porous Titanium Metaphyseal Sleeves*

**Figure 8:**
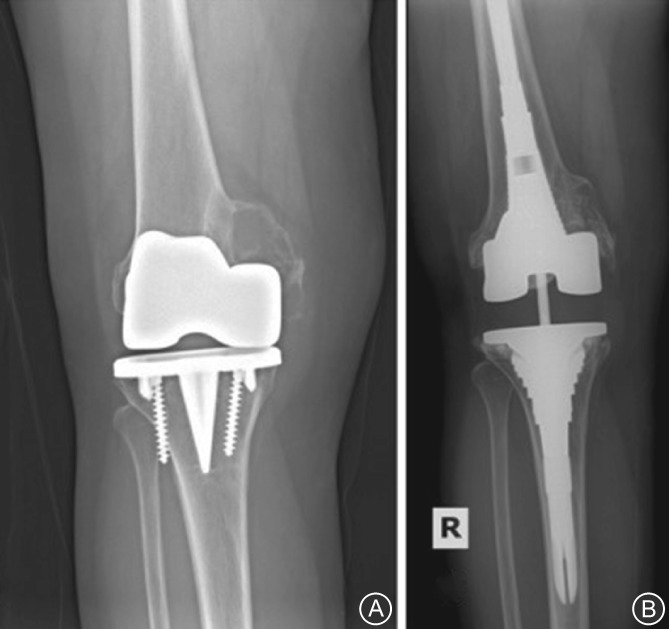
(A) Preoperative anteroposterior (AP) radiograph showing a Type IIA Anderson Orthopedic Research Institute tibial defect. (B) Postoperative AP radiograph (33 months) showing a cementless metaphyseal sleeve and stem construct.

Recently, porous titanium coated press fit metaphyseal sleeves have been developed to enhance fixation in managing the tibial defects during RTKA^35^. The interface between the metaphyseal sleeves and the implant is created by a Morse taper junction^15^. Metaphyseal sleeves are recommended for all tibial and femoral bone defects of AORI type 2 and 3^36^. Alexander *et al*. used a porous titanium tibial sleeve for AORI type 2B and 3 defects in RTKA patients with a minimum 2‐year follow‐up. The average KSS increased from 55 preoperatively to 92 postoperatively^37^. Their short‐term results showed that cementless metaphyseal fixation with sleeves is a promising option for the management of large defects and provides construct stability. Huang *et al*. used 36 metaphyseal sleeves in femoral revisions and 83 metaphyseal sleeves in tibial revisions and followed patients for an average of 2.4 years (range, 2–3.7 years)^54^. At final follow up, only 2 (2.7%) tibial components required revision for aseptic loosening. The mean Knee Society function score, the mean Short Form 36 physical score, the mean Western Ontario score, and the McMaster Universities Arthritis Index were improved. This indicated reliable metaphyseal fixation in RTKA at short‐term follow‐up. Barnett *et al*. suggested metaphyseal sleeves as a versatile option for metaphyseal fixation in RTKA (Fig. 8)^35^. They used metaphyseal sleeves for AORI type 2 and 3 tibial defects in RTKA and observed satisfactory clinical outcomes at a 2‐year follow‐up. At final follow‐up, significant improvements in knee range of motion and KSS were observed postoperatively (*P* < 0.001). Radiographic review at final follow‐up revealed stable, osteointegrated components without component migration or clinically significant osteolysis.

### *Porous Tantalum Metaphyseal Cones*

**Figure 9:**
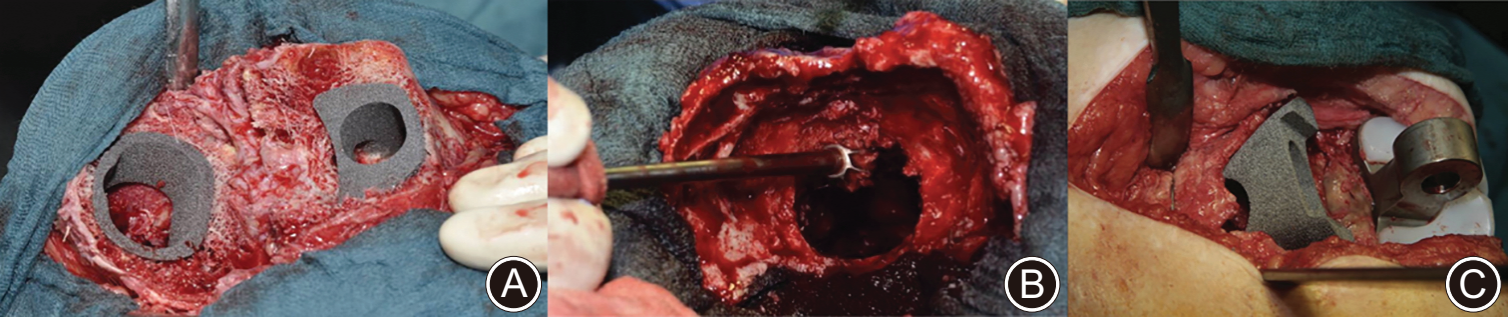
(A) Optimized sizing and *in situ* fixation of femoral and tibial cone. (B) Use of a high‐speed burr to ensure an optimal fit of the femoral TM cone. (C) Pressfit impaction of a femoral metal cone in combination with a hinged implant.

Porous tantalum structural cones are a relatively new indication for reconstruction of metaphyseal bone defects (AORI types 2 and 3) in RTKA^55^. Tantalum cones along with offset stems are implanted using the “press‐fit” system with maximum contact to the host bone and serve as a stable platform to provide structural and mechanical support^55^, ^56^. The use of porous tantalum structural cones is technically easier and has a lower risk of infection; however, they are difficult to remove and there is a risk of fracture of the host bone^38^. Schmitz *et al*. used porous tantalum cones in 44 patients and followed up for 37 months. the average preoperative KSS improved from 34 points (range, 6–90 points) to 63 points (range, 7–90 points) (Fig. 9)^39^. The VAS improved from 7.5 to 4.8. Only 2 patients required a re‐revision due to aseptic loosening. Their study showed favorable clinical and radiological outcomes in managing severe bone defects in RTKA. Meneghini *et al*. and Howard *et al*. report the early results of the use of a porous tantalum metaphyseal cone for severe tibial and femoral defects, respectively^57^, ^58^. Using the porous tantalum metaphyseal cone, both studies observed effective structural support for severe bone defects during RTKA.

## Megaprostheses/Customized Prostheses

**Figure 10:**
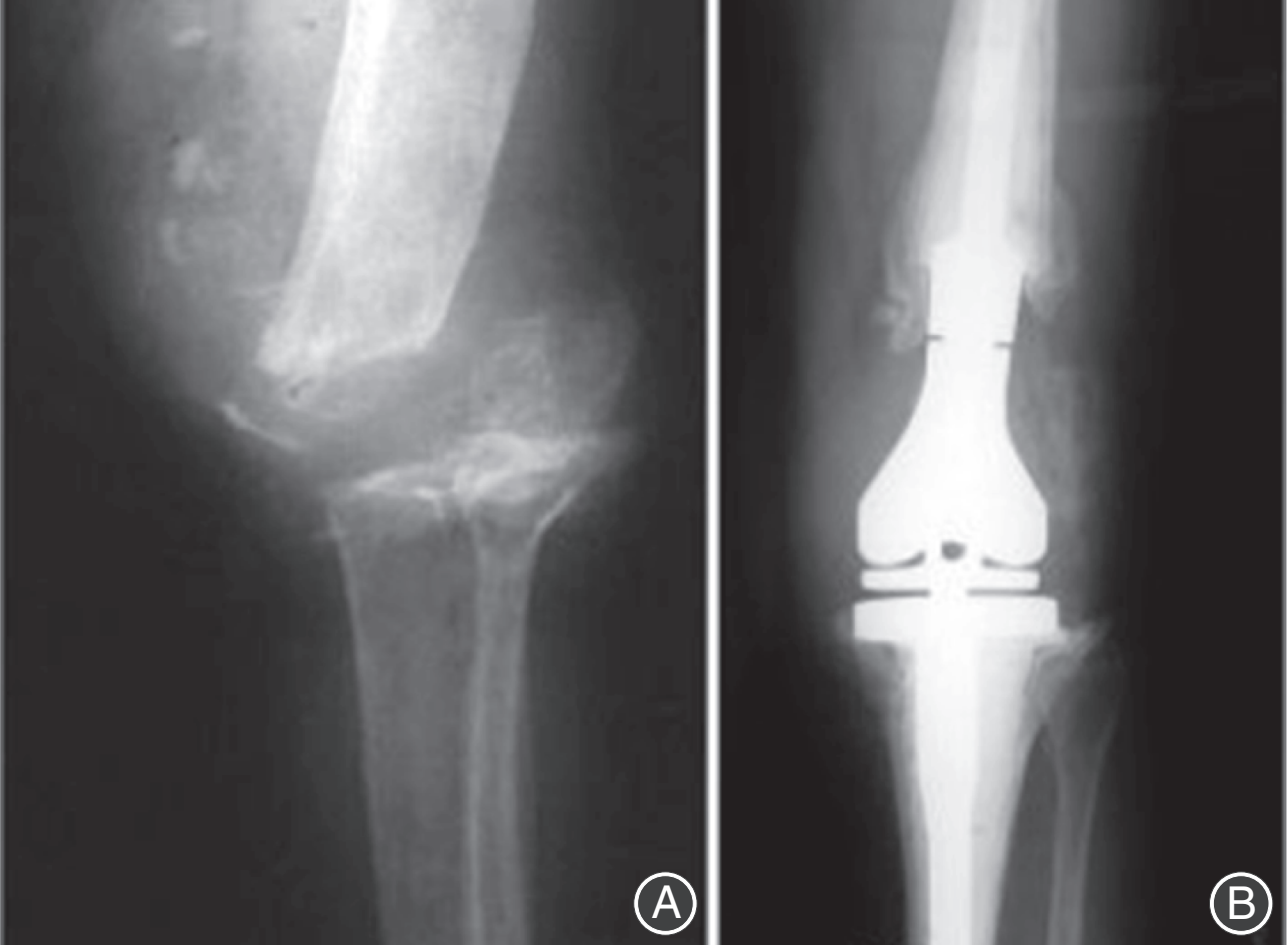
(A) Preoperative radiograph showing complex bone defects. (B) Postoperative anteroposterior radiograph showing treatment with a megaprosthesis with a rotating hinge device severe bone defects.

Megaprosthesis or customized prosthesis is an exceptional indication, which represents an excellent biomechanical solution for the management of complex bone defects in RTKA^59^. However, the customized implants are expensive, not versatile, take several weeks to manufacture, and have the risk of short‐term mechanical complications and infection often followed by amputation^15^. Fraser *et al*. suggested the use of megaprostheses with a rotating hinge device in salvage cases for severe bone defects, even though the revision‐free survival at 8 years is 58% with a total of 247 rotating‐hinge megaprostheses included in the study (Fig. 10)^60^. Höll *et al*. used megaprosthesis implantation in 20 non‐oncologic indications patients and followed up for 34 months (range, 10–84 months). Complications developed in 11 patients^59^. The KSS improved significantly, from 43 ± 15 to 68 ± 16.8; *P* < 0.05, indicating megaprostheses as a limb‐saving procedure which allows patients to avoid amputation and regain mobilization with full weight‐bearing in RTKA.

### *Conclusion*

**Figure 11:**
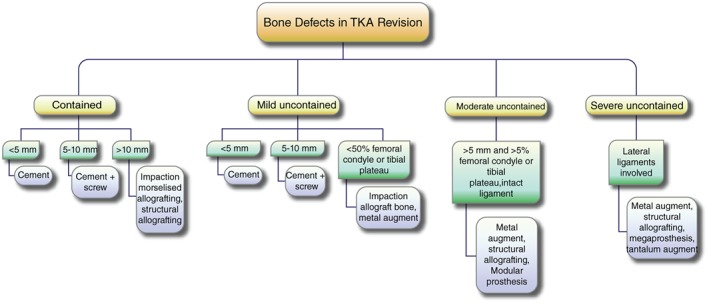
The summary of current management of the bone defects in revision of total knee arthroplasty.

Bone defects are commonly encountered during RTKA, which still has no standard treatment to cure. Only management of femoral and tibial bone defects is possible in some cases. The preference between different management systems depends on the patients and types of bone defects. Accurate diagnosis of bone defects and proper selection of treatment methods are necessary to improve the survival rate and construction stability. Currently, several techniques, instrumentation, biomaterials, and implant fixation have been developed to manage bone defects (Fig. 11). However, most of the management systems possess specific complications and unsatisfactory clinical outcomes. Novel approaches should be developed to improve the functional capacity, implant survival rates, and quality of life in a cost‐efficient manner.
